# Patient advocacy in tuberculosis research and treatment: an interview with Zolelwa Sifumba

**DOI:** 10.1242/dmm.052316

**Published:** 2025-03-19

**Authors:** Zolelwa Sifumba

**Affiliations:** Walter Sisulu University, Faculty of Medicine and Health Sciences, Mthatha, Eastern Cape, South Africa


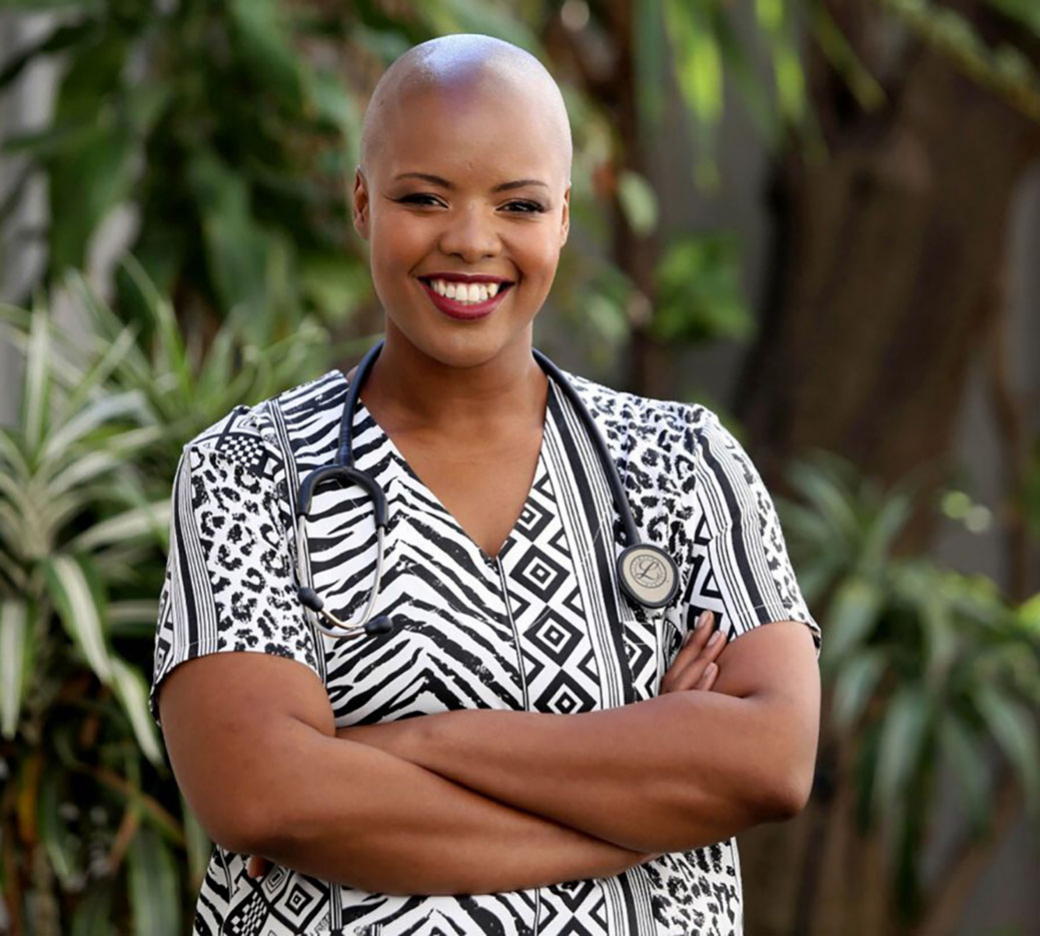


**Zolelwa Sifumba**

Multi-drug resistant tuberculosis (MDR-TB) is a severe form of TB caused by mycobacteria that are resistant to standard first-line treatment, typically isoniazid and rifampicin (Seung et al., 2015). In 2023, MDR-TB caused an estimated 150,000 deaths globally, and over half of the global number of patients with MDR-TB were based in five countries: India, Russia, Indonesia, China and the Philippines (WHO 2024). MDR-TB is one of the most-severe occupational hazards for healthcare workers worldwide, particularly in low and lower-middle income countries (Menzies et al., 1995; Fennelly and Iseman, 1999; von Delft et al., 2015, 2016). However, essential workers, who are most vulnerable to contracting TB, lack adequate protection in medical, social and psychological aspects from their hospitals and governments (https://www.civicus.org/index.php/media-resources/news/interviews/7132-south-africa-healthcare-workers-are-in-distress-too-and-we-need-action-now). As a result of this, a significant proportion of doctors and clinicians end up leaving their practice in many parts of the world (Elsevier's 2022 Report: Clinician of the Future). The Covid-19 pandemic had a devastating effect on TB care, research and funding. To this end, healthcare workers and advocates in Kenya and India reported a massive decline in the number of patients coming to TB health facilities (https://www.forbes.com/sites/madhukarpai/2020/09/26/tuberculosis-and-covid-19-fighting-a-deadly-syndemic/).

Dr Zolelwa Sifumba is a clinician, researcher and global health activist. She is a survivor of MDR-TB, which she contracted through occupational exposure while working long shifts as a medical student in Cape Town, South Africa. Zolelwa has worked at the Africa Health Research Institute as a clinical research fellow and is also a HBNU Fogarty Global Health Fellow (2022-23), through which she conducted TB research funded by the National Institute of Health (NIH), USA. In this interview, she discusses her personal experience of combating MDR-TB and her unwavering dedication to working as an activist and a patient advocate. Zolelwa highlights how her experience as a TB survivor, along with her training as a medical doctor, complements her work as an activist, and how the scientific and medical communities should work towards incorporating patient voices into disease research.

**Figure:**
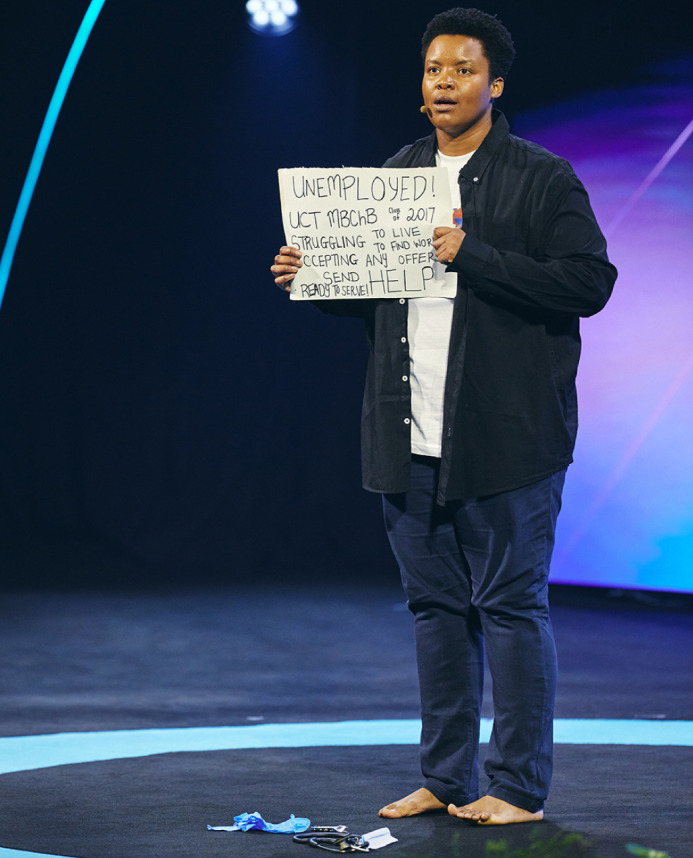
**Zolelwa Sifumba at the 2024 Skoll World Forum, a gathering of social innovators and leaders, in Oxford, UK**.

## What inspired you to originally train as a doctor and then eventually move into research. Did your own experience of surviving MDR-TB influence your career choices?

My first acquaintance with medicine was through my father, who was a doctor. As a kid, I didn't exactly understand a lot about what doctors do, but I saw that my father helped people. I was academically gifted since childhood, and I loved mathematics (and I still do). Consequently, my high school teachers kept advising me to get into lucrative careers, such as chartered accountancy. But I remember enjoying lessons in human biology at school. One day, I accidentally cut my finger and over the next few days, I observed how it healed. I remember appreciating how cool that was. Eventually, I started thinking about my career options. As the eldest daughter, I was used to taking care of people − it came very naturally to me. I also had exceptional academic score points that were required to qualify for admission into medical schools. So, when I received an offer letter from the University of Cape Town to pursue a Bachelor of Medicine and Surgery, I immediately took it. During my clinical placement at GF Jooste Hospital in Cape Town, I contracted MDR-TB – a form of TB that doesn't respond to therapeutic drugs. Soon, I understood that healthcare workers on the front lines are amongst the highest risk groups for acquiring MDR-TB – something that was never mentioned to us during our training. It was frustrating because our own university and hospital failed us. I had to undertake 18 months of intense treatment that involved toxic injectables because MDR-TB does not respond to milder therapeutics that are widely used against normal TB. The side-effects were horrible: I was nauseous and suffered from mild hearing loss and acute depression. The treatment even made me suicidal.

Later, nearly two years after earning my degree in medicine, I was posted in the rural settings of KwaZulu-Natal as a community service doctor. Working there as a clinician proved to be a tough experience. Junior doctors were frequently subjected to bullying behaviours and harassment from senior colleagues. The hospital infrastructure wasn't equipped to protect its healthcare workers from contracting severe infections. These circumstances made me struggle with anxiety and depression, and I got burnt out in the job. In 2020, when the world was prioritising infection prevention and control, my hospital wasn't taking this subject seriously, and I routinely read about healthcare professionals dying on the front line. This was when I felt like the system was failing us once again. Soon, my work as an activist and an advocate started to conflict with my profession as a clinician because I was still a part of the system. I was also bullied by senior colleagues for speaking out. Eventually, I left clinical practice because it wasn't sustainable for me anymore, and I moved to Johannesburg. At this point, I had already been an activist in the space of infectious diseases for a couple of years, through which I had networked with several scientists and researchers. At the end of the day, research informs policy changes and I wanted to be involved in the ‘behind-the-scenes’ TB research. I wanted to understand how scientists decide what avenue to focus on, how therapeutics are developed, how projects are funded and who decides on the funding. I thought my standing as a TB survivor and a clinician made me valuable in the realm of TB research. My aim was to make TB research more accessible to civil society, so that patients could directly benefit from ongoing progress. The HBNU Fogarty Global Health Fellowship gave me the opportunity to conduct research on subclinical TB for one year. This was followed by another year of research funded by the Africa Health Research Institute and six months of clinical trials in the Clinical HIV Research Unit at the University of Witwatersrand, Johannesburg. I am pleased to say that this work has recently been published by BMC Global and Public Health (Sifumba et al., 2024).

## I can only imagine how difficult it must be to publicly address your own journey of being a TB survivor. May I ask when and why you first decided to voice your testimony?

The courage came from seeing Dr Dalene von Delft, an MDR-TB survivor and founder of TB Proof (Box 1). Dalene and her husband came to my medical school to talk about TB awareness and patient advocacy after I had been diagnosed with the disease. It was a special experience because I needed to be in touch with someone who had dealt with the disease firsthand. I met with the speakers immediately after the session and shared my own experience. Dalene was extremely compassionate and took me in as a friend. They also invited me to speak at their next session in the university. This was the first time I got on stage and shared my story with my peers. I remember how everyone listened, almost in disbelief, that I, a fellow medical student, was battling MDR-TB and enduring the painful treatment process. It was a moment of extreme vulnerability, but it also helped me realise the power of my voice for the first time. After that day, I became increasingly more involved with advocacy work within the institute, and I saw how it encouraged more students to come out and speak about their own journeys in battling with TB. Earlier we used to be sent to the TB wards without proper protection, such as N95 respirators; however, after I began advocating for proper care and support for healthcare workers in our medical school, many of my junior colleagues refused to step into the hospital without proper personal protective equipment. These were some of the initial instances that made me believe in the power of activism and in raising my voice against systemic issues.

**Box 1. TB-Proof**

TB Proof is non-profit TB advocacy organisation based in South Africa that was founded by health care workers and students in 2012. The sole mission of this organisation is to “combine stories and science to make the world TB proof” (see https://tbproof.org/who-we-are/). Armed with staff members who have experienced the wrath of TB firsthand, TB Proof strives to build advocacy among TB affected communities and provides opportunities so that patients can directly inform TB research and care. This organisation has created a platform that mobilises resources for TB prevention through activism and community outreach. Their mission implements steps to make healthcare facilities, including hospitals, clinics and hospices safe for healthcare workers, while destigmatising all forms of TB (latent and active infection, MDR-TB and extensively drug-resistant TB).

TB Proof members are spread all over the world and they consistently advocate for increasing TB awareness (https://tbproof.org/using-science/). In 2019, TB Proof members Zolelwa Sifumba and Phumeza Tisile met with several world leaders and affected community representatives to demand an increase in research funding for TB, HIV and malaria at several global meetings and symposiums, including the Global Fund 6th Replenishment.

## As an MDR-TB survivor, a doctor, TB care advocate and a researcher, how do you think incorporating advocacy views into research in infectious diseases can benefit both patients and the research itself? Also, how do you think the current scenario is in connecting patients to mainstream research?

Throughout my work as an activist, I have witnessed researchers making a big difference in TB therapeutics and patient care. I have seen major policy shifts due to new evidence that surfaced in the field, which highlights the role of researchers in influencing major changes. As an outspoken activist, I often find that, while many people agree with my views, this doesn't always lead to significant policy changes. It is crucial to set research priorities that are based on patients' voices, because they understand the gaps in current therapeutics better than anyone else. I hope that policies informed by survivors' experiences would have a greater impact over time.

Patients, doctors and researchers need to work in collaboration to truly advance the field of disease research and bring impactful policy changes

When I am invited to speak at science conferences, my opportunity to advocate for patient-centred approaches is often limited. While mainstream researchers are allocated prominent slots in the space, I am usually relegated to smaller community-focussed panels. Survivors like me rarely feature in plenary sessions where high-profile discussions take place. This imbalance is concerning. The perspective of researchers who haven't experienced the disease firsthand often overshadow those of survivors. Even when I am granted a limited slot to speak at a medical conference or seminar, I share valuable personal insights with the audience, which many of the academics who haven't suffered the disease won't know. For instance, I candemonstrate how amikacin, a drug commonly used to treat MDR-TB, causes tremendous body pain, which highlights the need for research to develop better alternatives. Although we have come a long way in respect to increasing patients' involvement in disease research policies compared to my days as a junior doctor, we still need to do better as a society. When I enter a room as a doctor instead of a survivor, people tend to listen to me more – this mentality needs mending. Patients, doctors and researchers need to work in collaboration to truly advance the field of disease research and bring impactful policy changes.

## Can you think of any specific examples where advocates have helped set research priorities for TB research?

When I was undertaking TB treatment, the injectables used to cause tremendous pain along with hearing loss and nausea. I couldn't eat anything for days. So, there was a significant push in the civil society to replace the injectables with a new drug called bedaquiline. Although the FDA had approved this drug, the process of finalising its launch was being delayed due to unknown political or business reasons. To stand up against this lag in releasing the drug that was going to be such a game changer for patients, a group of fellow advocates and I protested at the South Africa TB conference. It was a surreal experience to see the passionate community of survivors and patients, who held posters and placards to protest, while I spoke to the audience about the excruciating side-effects of the few MDR-TB therapeutics that existed in the market back then. Although Dalene brought bedaquiline to the market in South Africa for compassionate use, the push to make this drug a major part of the Department of Health guidelines for the public health system came from the combined efforts of advocates and activists.

Another example is the impact that patient advocacy has had in influencing the platforms for patient support and care. When I was diagnosed with TB, the government or the hospital didn't assist me financially, although I had contracted the disease through my work as a public healthcare worker. It was a struggle to manage my finances while undergoing the severe treatment plan. Recently, the unwavering work of TB advocates and organisations, such as TB Proof (https://tbproof.org/), to implement social compacts forced the policy makers to dispense financial stipends and social support for patients undertaking TB treatment.

These are changes that have been made possible by the survivors' community and I want to applaud us on that.

## When it comes to clinical trials for infectious diseases, like MDR-TB, do you think people from low- or middle-income countries are underrepresented?

I think there have been global strides to involve the historically under-represented communities in mainstream clinical research and trials, and I am optimistic that things are improving. I have worked in stellar research institutes in South Africa and, so, I know that we are progressing as a nation. However, a lot of work is still needed to attain equitability. Having medical research centres doesn't mean that the local populations in low-income and lower-middle-income countries are involved in the decision-making process for research and development. So, the scale of representation of people from different communities needs to be expanded. Often, I witness the culture of tokenism when it comes to inviting speakers from underrepresented backgrounds to international conferences. Additionally, there are other systemic factors and bureaucracy that prevent individuals of certain nationalities from being able to secure proper travel documents/visas for attending a conference per se. So, I think it's important to investigate and acknowledge these societal disadvantages so that we can take proper steps to incorporate true inclusivity within the research community.

## How would you like to see the field of infectious disease research evolve/ what do you think the priorities should be in the field?

I envision the field to be more community-based in the future so that it is truly representative of patient and survivor groups. In the last decade, I have witnessed the power of storytelling. Listening to individuals who have been directly or indirectly affected by a disease helps in humanising the field of disease research, which would then translate into efficient research for developing better therapeutics. Recently, I was invited to a conference on basic disease biology in Germany with approximately a hundred attendees. When asked how many of them directly interact with patients to understand which research topics need attention, only four or five people raised their hands. So, I tried to reinstate the importance of meeting real people who are struggling with the disease, instead of staying ignorant within the confinements of textbook knowledge. I know there is a lot of fear around infectious diseases, which is why we need more advocates to empower and educate, including researchers and clinicians, rather than succumbing to fear-based tactics. The way to overcome these norms and prejudices would be to focus on person-centred research. In addition to highlighting acute diseases, where there is a possibility of completing treatment and surviving, we should also talk about individuals diagnosed with chronic long-term conditions, such as HIV. Involving activists, patients, survivors, their families within the active circle of research and policy making is an absolute uncompromisable necessity.

We dream about the day our voices and work would receive the spotlight that researchers and clinicians share!

When I was going through the TB treatment, being within the hospital premises with doctors and specialists all day used to make me extremely overwhelmed and depressed, to the point that I lost my will to live. But having a community of TB survivors and patients around me was the biggest support system during those difficult times. Sharing our experiences and tips for managing everyday struggles kept us alive. The relatability that comes from shared ‘tips and tricks’ weren't something that we could acquire in the hospital from professional doctors. It baffles me that, as a society, we are not utilising the full potential of patient perspectives and feeding them into research and policy making. That would, indeed, make true difference. I have also talked about the need for mental health support during and after TB treatment and care (Schoeman and Sifumba, 2021). I dream that all these disparate communities: researchers, advocates, patients and survivors, clinicians and policy makers, would interact within the same ecosystem to push the boundaries of disease research. Many medical journals publish data on a disease or a clinical trial that are driven by samples from the patient community; however, the skyrocketing journal subscription charges and inaccessible scientific language prevent patients and advocates from appreciating advances in [treating] the disease that affects them. It's hypocritical and extremely unfair. That's why we need more science outreach programmes and engagement of the research community with the lay crowd. As activists, we love the work we do. And we dream about the day our voices and work would receive the spotlight that researchers and clinicians share!

## Conclusions

Organisations like TB Proof and individuals such as Zolelwa Sifumba have aided in the evolution of patient advocacy in disease research at an international level. This involves a positive push towards patient empowerment by directly involving TB patients in cutting-edge TB research. While treatment for TB itself involves a long and challenging period, often lasting more than 6 months, MDR-TB comes with its own added of challenges. According to the World Health Organisation (WHO), MDR-TB's diagnoses and treatment remain sub-optimal on a global level with less than one-third of the patients being properly diagnosed and only about 5% successfully completing treatment (Kurz et al., 2016). In this interview, Zolelwa shares her experience as an MDR-TB survivor, a clinician and researcher and an advocate and activist. She outlines how researchers and policy makers must work alongside patients to accelerate research in disease biology. Indeed patient advocacy can inform research in TB and other infectious diseases (both acute and chronic) in unique ways that can massively improve the pace and quality of diagnoses, treatment and preventative care in the future.
